# Life cycle assessment of microalgal cultivation medium: biomass, glycerol, and beta-carotene production by *Dunaliella salina* and *Dunaliella tertiolecta*

**DOI:** 10.1007/s11367-023-02209-2

**Published:** 2023-07-24

**Authors:** Gleison de Souza Celente, Rosana de Cassia de Souza Schneider, Jennifer Julich, Tiele Medianeira Rizzetti, Eduardo Alcayaga Lobo, Yixing Sui

**Affiliations:** 1 Environmental Technology Post-graduation Program, University of Santa Cruz do Sul, Avenida Independência, 2293, Santa Cruz do Sul, Rio Grande do Sul, 96815-900 Brazil; 2 Centre of Excellence in Oleochemical and Biotechnological Products and Processes, University of Santa Cruz do Sul, Avenida Independência, 2293, Santa Cruz do Sul, Rio Grande do Sul, 96815-900 Brazil; 3 School of Science, Faculty of Engineering and Science, Central Avenue, University of Greenwich, Chatham Maritime, Kent, ME4 4TB UK

**Keywords:** Life cycle assessment, *Dunaliella salina*, *Dunaliella tertiolecta*, Modified Johnson medium, Medium optimization, Carbon footprint

## Abstract

**Purpose:**

*Dunaliella* is a halophilic genus of microalgae with high potential in the global food market. The microalgal cultivation process contributes to not only economic impact but also environmental impact, especially regarding the artificial medium composition. In this context, a life cycle assessment was carried out to analyze the impacts associated with the components of the modified Johnson medium (MJM) and to predict the best scenarios to cultivate *Dunaliella tertiolecta* and *Dunaliella salina* for biomass, glycerol, and beta-carotene production.

**Method:**

Two chains were analyzed separately: (1) *Dunaliella salina* (strain DF 15) cultivated in 8 scenarios combining different nitrogen (0.1 and 1.0 g L^−1^ KNO_3_) and magnesium (1.1–2.3 g L^−1^ MgCl_2_.6H_2_O) concentrations to produce biomass, glycerol, and beta-carotene and (2) *Dunaliella tertiolecta* (strain CCAP 19/30) cultivated in 5 scenarios combining different nitrogen (0.1 and 1.0 g L^−1^ KNO_3_) and salt (116.9–175.4 g L^−1^ sea salt) concentrations to produce biomass and glycerol. In addition, we evaluated the potential of cultivating these species to reduce the carbon footprint of the proposed scenarios.

**Results and discussion:**

For *D. salina*, S_5_ (1 g L^−1^ KNO_3_, 1.1 g L^−1^ MgCl_2_.6H_2_O) had the lowest environmental damage for biomass (74.2 mPt) and glycerol production (0.95 Pt) and S_3_ (0.1 g L^−1^ KNO_3_, 1.9 g L^−1^ MgCl_2_.6H_2_O) for beta-carotene (3.88 Pt). T_4_ (1 g L^−1^ KNO_3_, 116.9 g L^−1^ sea salt) was the best for *D. tertiolecta* for biomass (74 mPt) and glycerol (0.49 Pt). “Respiratory inorganics,” “Non-renewable energy,” and “Global warming” were the most impacted categories. “Human health,” “Climate change,” and “Resources” had the highest share of all damage categories. All the scenarios presented negative carbon emission after proposing using brine as alternative salt source: S_5_ was the best scenario (− 157.5 kg CO_2_-eq) for *D. salina* and T_4_ for *D. tertiolecta* (− 213.6 kg CO_2_-eq).

**Conclusion:**

The LCA proved its importance in accurately predicting the optimal scenarios for MJM composition in the analyzed bioproducts, as confirmed by the Monte Carlo simulation. Although the absolute values of impacts and productivity cannot be directly compared to large-scale cultivation, the validity of the LCA results at this scale remains intact. Productivity gains could outweigh the impacts of “surplus” MJM components. Our study showcased the potential of combining *D. salina* and *D. tertiolecta* cultivation with CO_2_ capture, leading to a more environmentally friendly cultivation system with a reduced carbon footprint.

**Supplementary Information:**

The online version contains supplementary material available at 10.1007/s11367-023-02209-2.

## Introduction

Microalgae have a long history of being proposed as a food source (Kay and Barton 1991). They offer an attractive alternative to conventional land plants due to their high protein content and less land requirement for their cultivation (Vanthoor-Koopmans et al. 2013). Microalgae are also known to produce valuable bioproducts for applications in the food industry (Dufossé et al. 2005; Matos 2017), such as glycerol (Monte et al. 2020) for emulsifiers, shorteners (Morrison 2000), and edible food packaging (Atta et al. 2022), and pigments for natural coloring (Dufossé et al. 2005). *Dunaliella* is a halophilic genus worldwide known for its pharmaceutical and nutraceutical benefits owing to the production of active compounds (de Souza Celente et al. 2022). Their biomass use is commonly associated with obesity control (Melnikov et al. 2022), anti-inflammatory (Wang et al. 2022), and anti-cancer (Chen et al. 2021) activity. Currently, their use for human consumption is still limited in Europe. Yet, its generally recognized as safe (GRAS) status granted by the US Food and Drug Administration (US Food & Drug Administration 2020) and wide acceptance in Asia as a conventional food source indicate their promising global market potential. In 2020, the food and pharmaceutical sectors shared approximately 75% of the global *D. salina* market share, i.e., 67.9 M USD (Maia Research 2021).

Along with cultivation cost (Colusse et al. 2020) and restrictive/non-existent legislation (Harvey and Ben-Amotz 2020), consumer acceptability is a major bottleneck for commercializing microalgae-based products (Nova et al. 2020). This consumer behavior, known as food neophobia (i.e., repulsion for non-traditional food), can make it difficult for novel food products to be introduced to the market (García-Segovia et al. 2020). Fortunately, the scenario for microalgae as food has been steadily improving (García-Segovia et al. 2020) as “green labeling” largely aids in publicizing the benefits of microalgae consumption for human health and the environment. Nonetheless, such “green” marketing can also be misleading (Ihemezie et al. 2018). For instance, the cultivation process, especially regarding the usage of artificial medium, can contribute to a great environmental impact due to the consumption of non-renewable resources, such as fossil energy, water, and macronutrients (Chen et al. 2015). Thomassen et al. (2018) evaluated the environmental impact of different hypothetical large-scale scenarios to cultivate *D. salina* for beta-carotene, and cultivation had the greatest impact among all the steps analyzed: cultivation, pre-harvest, harvest, washing, drying, extraction, and purification. Unlike unconventional cultivation media such as wastewater, artificial and semi-artificial media using natural water and artificial nutrient supply offer better-controlled and replicable conditions for microalgae cultivation (Bauer et al. 2021). In this way, microalgal biomass and bioproduct productivities are often increased, which is a reliable approach to lessening the environmental, social, and economic impacts of artificial media (Chen et al. 2015; Bauer et al. 2021; Kabir et al. 2022). It can be expected that improving microalgal biomass and bioproduct productivities by optimizing the cultivation media could potentially reduce the environmental impacts.

Unlike an economic analysis, evaluating a certain process’s environmental and social impact is not straightforward, requiring a more complex and dedicated tool. Life cycle assessment (LCA) is a widely accepted approach to categorizing environmental loads based on inputs and outputs. It helps to compare different scenarios to identify the best method for a common issue/goal and processes that still need improvement (Guinée 2002). LCA converts different aspects (inputs and outputs) into easier-to-interpret data, facilitating decision-making (Sun et al. 2019). However, LCA of *Dunaliella* sp*.* cultivation is addressed by only a few papers (e.g., Thomassen et al. (2018) and Keller et al. (2017)). So far, this is the first approach addressing different artificial medium compositions to optimize bioproduct yield by two *Dunaliella* species and improve the environmental aspect. In this context, an LCA was conducted to analyze the impacts of using artificial medium and predict the best scenarios to cultivate *D. salina* and *D. tertiolecta* for biomass, glycerol, and beta-carotene production on a laboratory-scale. Two chains were analyzed separately: (1) *D. salina* cultivated in eight scenarios combining different nitrogen and magnesium concentrations to produce biomass, glycerol, and beta-carotene, and (2) *D. tertiolecta* cultivated in five scenarios combining different nitrogen and salt concentrations to produce biomass and glycerol. Since *D. tertiolecta* is not carotenogenic, beta-carotene was not assessed for this species. In addition, the potential for carbon footprint reduction following this LCA has been evaluated, and the hypothetical use of brine from a desalination plant was analyzed.

## Methodology

### LCA goal, scope, and boundaries

A gate-to-gate assessment was performed for the cultivation of *D. salina* DF 15 and *D. tertiolecta* CCAP 19/30 to produce biomass, glycerol, and beta-carotene (only for *D. salina*) following ISO 14044 (2006) guidelines. The life cycle impact analysis (LCIA) was performed using SimaPro software version 8.5 considering 1 kg of biomass/bioproduct produced (functional unit). The method chosen for the LCIA was Impact 2002+ (Humbert et al. 2012). Table 1 shows the impact categories and the equivalent unit. The impact analysis was shown as normalized and non-normalized results. In normalized results, a value is attributed to each category quantifying the respective share to the overall damage. In non-normalized results, the value of 100% is attributed for a specific category to the scenario with the highest impact, and for the remaining scenarios, the impact is calculated relative to the former scenario. Only impact categories with values greater than 1 × 10^–3^ (after normalization) were selected for the figures and tables in the result section to improve readability (except for the Monte Carlo simulation), as their contribution was irrelevant compared with the other categories (the complete list of categories is shown in Table 1).

**Table 1:** Analyzed impact categories, their equivalent units, and resulting damage categories using Impact 2002+ (SimaPro 2020)

Impact categories	Unit	Damage categories
Carcinogens	kg C_2_H_3_Cl eq	Human health
Non-carcinogens	kg C_2_H_3_Cl eq	
Respiratory inorganics	kg PM_2.5_ eq	
Respiratory organics	kg C_2_H_4_ eq	
Ionizing radiation	Bq C_-14_ eq	
Ozone layer depletion^*^	kg CFC_-11_ eq	
Aquatic ecotoxicity	kg TEG water	Ecosystem quality
Terrestrial ecotoxicity	kg TEG soil	
Terrestrial acidification/nutrification	kg SO_2_ eq	
Land occupation	m^2^ org.arable	
Aquatic acidification	kg SO_2_ eq	
Aquatic eutrophication	kg PO_4_ P-lim	
Global warming	kg CO_2_ eq	Climate change
Non-renewable energy	MJ primary	Resources
Mineral extraction	MJ surplus	

^*^Also accounting for the “Ecosystem quality” damage category

Each chain (*D. salina* and *D. tertiolecta* cultivation) was assessed individually for its proposed scenarios (Tables 2 and 3) through a specific inventory of input/output, as the goal was to compare the scenarios within each chain. The LCA included only the impacts associated with the production of chemicals for the artificial medium (modified Johnson medium (MJM) (Borowitzka (1988); see Table 4 for details), retrieved directly from Ecoinvent 3.6 and Agri-footprint databases; the contribution of infrastructure and electricity for the equipment used for cultivation, biomass recovery, and bioproduct extraction were not included in this scope.

**Table 2:** Different scenarios for *D. salina* cultivation and their respective inputs and outputs

Inputs/outputs	Scenarios							
	S_1_	S_2_	S_3_	S_4_	S_5_	S_6_	S_7_	S_8_
*Concentration of varied MJM components*								
KNO_3_	0.1	0.1	0.1	0.1	1	1	1	1
MgCl_2_.6H_2_O	1.1	1.5	1.9	2.3	1.1	1.5	1.9	2.3
*Productivity*								
Biomass (mg L^−1^ day^−1^ AFDW)	58	58	67	63	108	84	108	85
Glycerol (mg L^−1^ d^−1^)	5.31	5.71	7.94	6.09	8.21	8.05	8.47	5.86
Beta-carotene (mg L^−1^ d^−1^)	3.43	3.93	3.96	3.94	2.06	1.65	1.89	1.30

*D. salina* (3.3–3.4 × 10^4^ cell mL^−1^ initial cell density) was cultivated in 50-mL Erlenmeyer flasks containing 20 mL of MJM with the proposed modifications. The flasks were kept in a temperature-controlled chamber (Varicon Aqua, Worcester, UK) at 25 °C and approximately 100-µmol photons m^−2^ s^−1^ continuous LED white light for 18 (S_1_–S_4_) or 25 (S_5_–S_8_) days. Data were obtained from our previous (unpublished) experiments

**Table 3:** Different scenarios for *D. tertiolecta* cultivation and their respective inputs and outputs

Compounds (g L^−1^)	Scenarios				
	T_1_	T_2_	T_3_	T_4_	T_5_
*Concentration of varied MJM components*					
KNO_3_	0.1	0.1	0.1	1	1
Sea salt	116.9	175.4	233.9	116.9	175.4
*Productivity*					
Biomass (mg L^−1^ day^−1^ AFDW)	88	86	60	133	114
Glycerol (mg L^−1^ d^−1^)	4.28	6.26	7.39	20.28	19.32

*D. tertiolecta* (6.6–6.7 × 10^4^ cell mL^−1^ initial cell density) was cultivated in 50-mL Erlenmeyer flasks containing 20 mL of MJM with the proposed modifications. The flasks were kept in an Algem^®^ HT24 photobioreactor (Algenuity, Stewartby, Bedfordshire, UK; https://www.algenuity.com/; accessed October 24, 2022) at 25 °C; 200-µmol photons m^−2^ s^−1^ continuous LED white light, and 100 rpm agitation for 16 (T_1_–T_3_) or 18 (T_4_ and S_5_) days. Data were obtained from our previous (unpublished) experiments

**Table 4:** Chemical composition of the modified Jonhson medium and its substitute when necessary

Compounds	Concentration (g L^−1^)	Substitute in Ecoinvent or Agri-footprint databases
KH_2_PO_4_	0.035	Na_3_PO_4_
MgSO_4_.7H_2_O	0.5	
CaCl_2_.2H_2_O	0.2	
MgCl_2_.6H_2_O	1.5	MgSO_4_.7H_2_O and KCl
KCl	0.2	
KNO_3_	1	
NaHCO_3_	0.84	K_2_CO_3_
Sea salt	87.7	NaCl
FeCl_3_.6H_2_O	0.00244	
Na_2_EDTA.2H_2_O	0.00189	
H_3_BO_3_	0.00061	
MnCl_2_.4H_2_O	0.000041	
ZnCl_2_	0.000041	ZnO
CuSO_4_.5H_2_O	0.00006	
CoCl_2_.6H_2_O	0.000051	
(NH_4_)6Mo_7_O_24_.4H_2_O	0.00038	MoO_3_.nH_2_O

### Lifecycle inventory (LCI)

The LCI data was derived from the Ecoinvent 3.6 and Agri-footprint databases, and the inputs and outputs were obtained from laboratory-scale experiments. Some components in the medium were not found in the LCA databases, requiring replacement by similar compounds (Table 4). Scenarios within the same chain were cultivated using the same cultivation equipment; thus, energy and infrastructure inputs were disregarded for better visualization and comparison between scenarios. Furthermore, as the cultivation was done on a laboratory-scale, the energy impact would be overestimated and would not reflect what would happen on a large-scale. The only variables were the chemical composition of the medium and productivity. The LCA was limited to the composition of the artificial medium and productivity influence. As will be discussed in the third section, NaCl (substitute of sea salt in the inventory) had a major contribution to most of the analyzed impact categories; thus, to validate this LCA results, the hypothetical use of brine from desalination plants was analyzed (provided in the Supplementary Material).

### Carbon footprint

“Global warming” contribution was further discussed to identify the possibility of using the two species’ biomass to fix carbon to compensate for the CO_2_-eq emissions of the proposed scenarios and reduce the carbon footprint associated with cultivation in an artificial medium. It was considered that CO_2_ could replace NaHCO_3_ without changing the biomass yield based on our previous experiment (Celente et al. 2022). A 40% carbon content based on dry weight (DW) and a 1.83 g CO_2_ captured per g of biomass ratio were hypothesized (Acién Fernández et al. 2012). The hypothetical assumption of replacing sea salt with brine from desalination plants was proposed and analyzed for the best-predicted scenarios (S_3_, S_5_, and T_4_) to quantify the possible reduction in CO_2_ emission and damage categories.

### Data processing

**Fig. 1:**
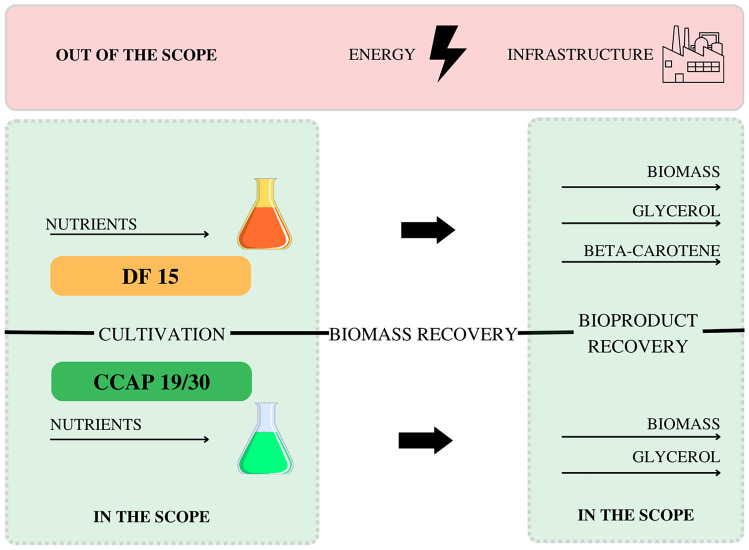
Steps for obtaining the target bioproducts of *D. salina* and *D. tertiolecta*. Dashed green rectangles demonstrate the boundaries of the “gate-to-gate” microalgae cultivation system for producing biomass, glycerol, and beta-carotene on a laboratory-scale. Solid red rectangles demonstrate the exclusions of the scope

Graphs were generated using OriginPro 2022 software (OriginLab Corporation, Northampton, MA, USA), excepted for Fig. 1, which was created using the Canvas website. Linear correlation (*r*) analysis was performed using the PAST v. 4.07 software (HAMMER et al. 2001) with a significance level of *p* ≤ 0.05 (all statistical requirements for using this parametric test were significantly proven). A positive linear correlation indicates that an increase in one variable results in an increase in the second; a negative linear correlation indicates that an increase in one variable results in a decrease in the second. Linear correlation coefficient (*r*) is an absolute value varying from − 1 to 1 (Taylor 1990), demonstrating the degree of linear association between two variables. Uncertainty analysis was performed using Monte Carlo simulation (normal distribution, 1000 interactions, and 95% confidence) for each category of the Impact 2002+ Method for the best scenarios for each product (biomass, glycerol, and beta-carotene) (McMurray et al. 2017).

## Results and discussion

### LCIA of the *Dunaliella salina* chain

#### Biomass

**Fig. 2:**
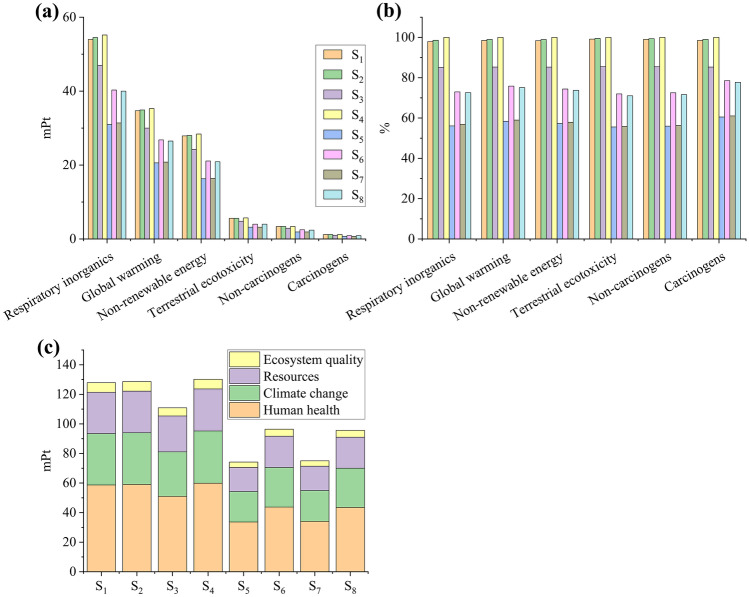
Normalized (**a**) and non-normalized (**b**) impact categories for each scenario and single score system for damage categories considering biomass produced by *D. salina*. Columns are sorted from S_1_ to S_8_ (**c**)

The greatest impact for *D. salina* cultivation was “Respiratory inorganics” (31.0–55.2 mPt), followed by “Global warming” (20.6–35.3 m Pt), and “Non-renewable energy” (16.3–28.4 mPt; Fig. 2a). NaCl (62–77%; Supplementary Material, “*D. salina* – Impact” tab) was the main contributor to the three mentioned categories. Thomassen et al. (2018) reported that salt and nutrients were the biggest contributors to environmental impacts in *D. salina* cultivation. Interestingly, supplementation with less nitrogen (S_1_–S_4_, 0.1 g L^−1^ KNO_3_) did not result in less impact: S_1_–S_4_ had its non-normalized impact ranging from 85.1 to 100% for all categories, while it varied between 55.9 and 78.5% for S_5_–S_8_ (1 g L^−1^ KNO_3_; Fig. 2b). S_3_ (0.1 g L^−1^ KNO_3_, 1.9 g L^−1^ MgCl_2_.6H_2_O; 111 mPt) was the best scenario among the lower nitrogen concentration group and S_5_ (1 g L^−1^ KNO_3_, 1.1 g L^−1^ MgCl_2_.6H_2_O; 74 mPt) between all scenarios. S_4_ was the worst scenario (0.1 g L^−1^ KNO_3_, 2.3 g L^−1^ MgCl_2_.6H_2_O; 130 mPt; Fig. 2a–c).

Biomass productivity (Table 2) may explain the damage results for scenarios S_5_–S_8_ (*r* =  − 1, *p* < 0.001) but not for S_1_–S_4_ (*r* =  − 0.78, *p* = 0.22), indicating that the biomass yields at S_5_–S_8_ were high enough to compensate the impact caused by using more nitrogen. Nitrogen concentration was negatively correlated with the damage result (*r* =  − 0.90, *p* = 0.002), which indirectly correlates with biomass productivity: higher yield at higher nitrogen concentration. Magnesium concentration did not have enough impact to influence the results (*r* = 0.08, *p* = 0.85): for instance, it contributed less than 7% to the impact categories for S_8_ where the highest amount of magnesium and nitrogen were used (Supplementary Material, “*D. salina* – Impact” tab). “Human health” had the highest share of all damage categories (34–60 mPt; Fig. 2c), which is a reflection of the “Respiratory inorganics” impact category (Table 1). Thomassen et al. (2018) reported that “Respiratory inorganics” contributed more to “Human health” when assessing different cultivation scenarios for growing *D. salina* for beta-carotene production. “Climate change” (Global warming) and “Resources” (Non-renewable energy) were the second and third largest damage categories. “Ecosystem quality” scored < 6.5 mPt as none of the proposed scenarios showed significant potential to impact any of the impact categories (e.g., “ozone depletion,” “aquatic toxicity,” and “Land occupation”) relevant to “Ecosystem quality.”

#### Glycerol

**Fig. 3:**
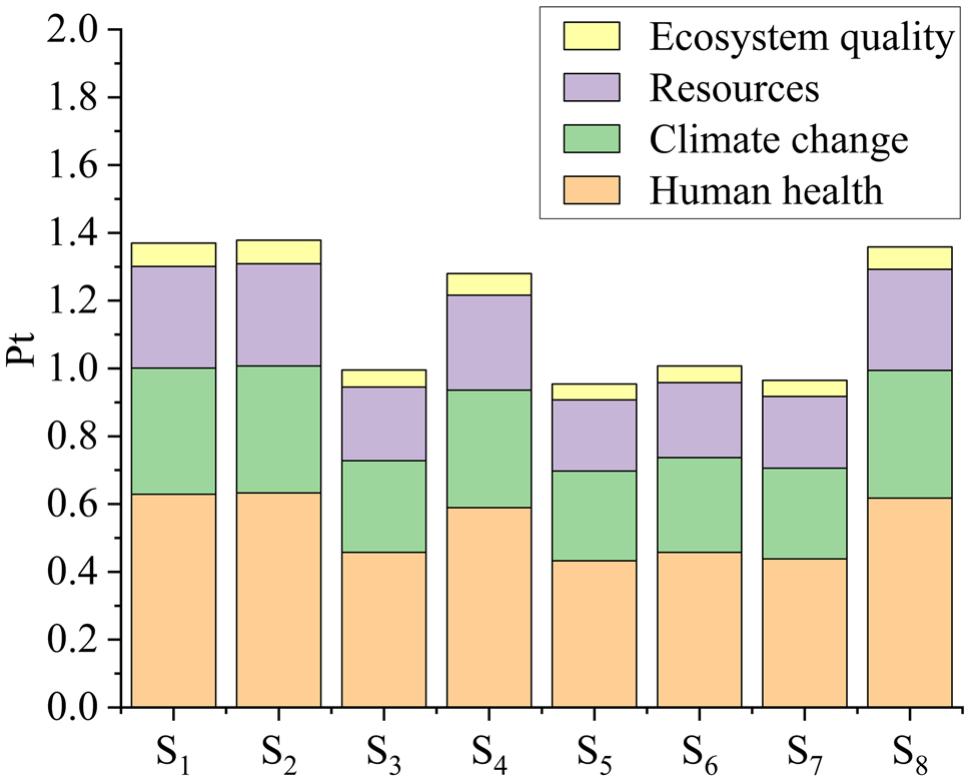
Single score system for damage categories for glycerol production by *D. salina*

*D. salina* is a good glycerol source, with productivity values between 5.31 and 8.47 mg L^−1^ d^−1^ (Table 2). Glycerol production is triggered to maintain the osmotic balance (Singh et al. 2019). The salt concentration (87.7 g L^−1^ sea salt) was the same for all *D. salina* scenarios; thus, glycerol production was proportional to biomass yield (*r* = 0.74, *p* = 0.04). As a result, the LCA result for glycerol was similar to that for biomass: lower nitrogen concentration induced lower biomass productivity (thus, lower glycerol yield), resulting in more impact/damage (Fig. 3). Among the scenarios S_1_–S_4_, S_3_ was the least impactful (1.00 Pt). Overall, the least impactful scenario was S_5_ (0.95 Pt). Unlike the biomass LCIA result, S_8_ (1.36 Pt) was the most impactful scenario, presenting the lowest glycerol productivity (1.30 mg L^−1^ d^−1^, Table 2). The glycerol productivity directly influenced the damage result (*r* =  − 0.99, *p* < 0.001), while the concentration of nitrogen (*r* =  − 0.50, *p* = 0.21) and magnesium (*r* = 0.16, *p* = 0.71) did not influence the results.

#### Beta-Carotene

**Fig. 4:**
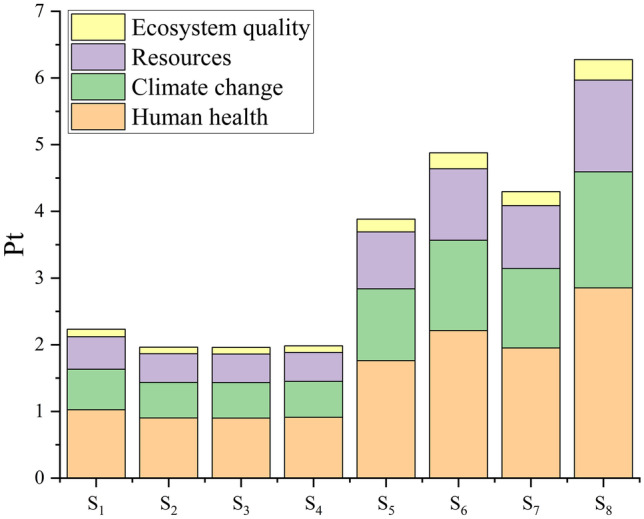
Single environment damage score for beta-carotene production by *D. salina*

Unlike the results from the biomass and glycerol analysis, the damage score demonstrates that the group with the lowest nitrogen concentration (S_1_–S_4_) had a smaller impact (2.0–2.2 Pt) than the group with the highest nitrogen concentration (S_5_–S_8_; 3.9–6.3 Pt; Fig. 4). Beta-Carotene production responds positively to nitrogen-limiting conditions (Han et al. 2019). Thus, S_1_–S_4_ offered the best condition for *D. salina* to produce it. S_8_ presented the worst scenario concerning beta-carotene due to its lower productivity (1.30 mg L^−1^ d^−1^ beta-carotene) and higher nitrogen (1 g L^−1^ KNO_3_) and magnesium (2.3 g L^−1^ MgCl_2_.6H_2_O) concentrations, while S_3_ (3.94 mg L^−1^ d^−1^ beta-carotene; 0.1 g L^−1^ KNO_3_; 1.9 g L^−1^ MgCl_2_.6H_2_O) performed better. Beta-Carotene productivity was 3.43–3.96 and 1.30–2.06 mg L^−1^ d^−1^ for the groups with the lowest and highest KNO_3_ concentrations, respectively (Table 2). Beta-Carotene showed a negative linear correlation with the single damage score (*r* =  − 0.97, *p* < 0.001). Nitrogen concentration presented a positive correlation (*r* = 0.91, *p* = 0.002), while magnesium and single damage score did not correlate (*p* = 0.21–0.61).

### LCIA of the *Dunaliella tertiolecta* chain

#### Biomass

**Fig. 5:**
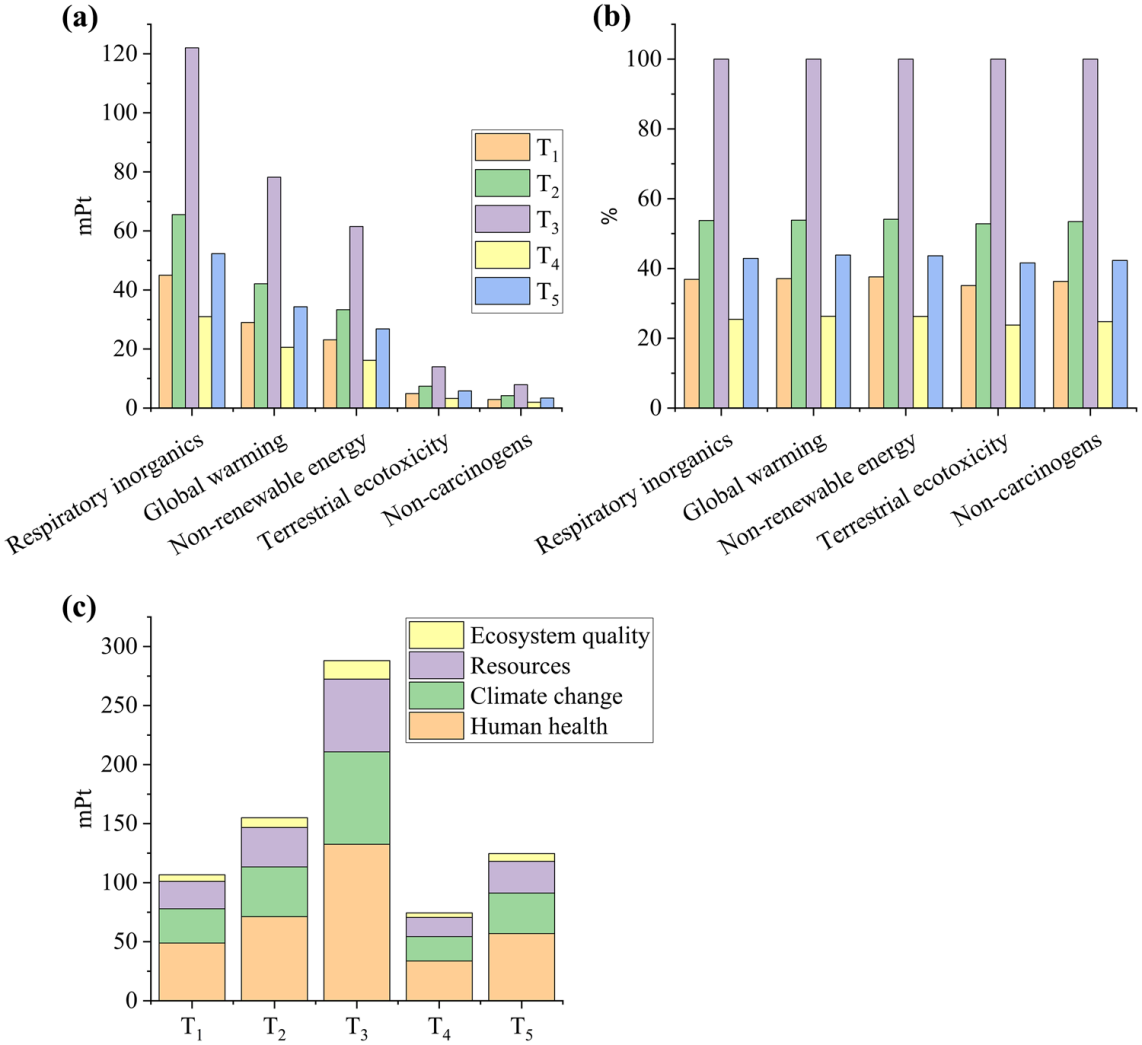
Normalized (**a**) and non-normalized (**b**) impact categories for each scenario and single scoring system for damage categories considering biomass yielded by *D. tertiolecta*. Columns are sorted from T_1_ to T_5_ (**c**)

The greatest impact for *D. tertiolecta* cultivation was “Respiratory inorganics” (31.0–122.0 mPt), followed by “Global warming” (20.6–78.2 mPt) and “Non-renewable energy” (0.02–0.06; Fig. 5a). NaCl was the main contributor to the three mentioned categories (77–91%; Supplementary Material, “*D. tertiolecta* – Impact” tab), which agrees with Thomassen et al. (2018). Although *Dunaliella* species can cope with hypersaline environments (> 150 g L^−1^ salt concentration), biomass yield can be significantly impaired at salt concentrations greater than 135 g L^−1^ (Ishika et al. 2019). In the same way as the results of *D. salina* cultivation, the supplementation of less nitrogen (T_1_–T_3_, 0.1 g L^−1^ KNO_3_) did not result in a smaller impact: T_1_–T_3_ had their impact varying between 35.1 and 100% for all categories, while it varied between 23.8 and 43.9% for T_4_ and T_5_ (1 g L^−1^ KNO_3_; Fig. 5b). T_4_ (1 g L^−1^ KNO_3_, 116.9 g L^−1^ sea salt; 74.4 mPt) was the best scenario, while T_3_ (0.1 g L^−1^ KNO_3_, 233.9 g L^−1^ sea salt; 288.1 mPt) was the worst (Fig. 5a–c).

Overall, T_4_ and T_5_ had 1.3–1.5 times more biomass than their counterparts (T_1_ and T _2_, respectively), directly impacting the LCA: biomass productivity and damage results were negatively but not significantly correlated (*r* =  − 0.85, *p* = 0.07). Nitrogen concentration did not affect the LCA (*r* =  − 0.55, *p* = 0.33). This was expected because KNO_3_ had a small share of the overall impact (< 9%, data not shown). Sea salt concentration, as expected, correlated positively with damage results (*r* = 0.93, *p* = 0.02). “Human health” (33.8–132.6 mPt; Fig. 5c), followed by “Climate change” (20.6–78.2 mPt), and “Resources" (16.2–61.5 mPt), presented the largest share of all damage categories. As identified for *D. salina*, “Ecosystem quality” (< 15.7 mPt) was the lowest damage category for *D. tertiolecta* cultivation.

#### Glycerol

**Fig. 6:**
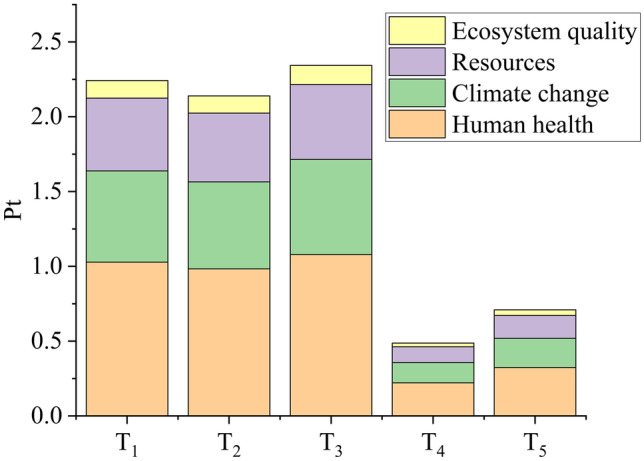
Single scoring system for damage categories for glycerol production by *D. tertiolecta*

Glycerol productivity varied between 4.28 (T_1_) and 20.28 mg L^−1^ d^−1^ (T_4_) for *D. tertiolecta* (Table 3). Its productivity results from glycerol content and biomass growth and is triggered to deal with osmotic shock (Singh et al. 2019; de Souza Celente et al. 2022). In our experiments, glycerol productivity and salt concentration correlated (*r* = 0.99, *p* = 0.1) for T_1_–T_3_. For T_4_ and T_5_, the correlation was impossible to calculate due to the limited amount of data; however, there was a small reduction in glycerol productivity at T_5_ due to lower biomass yield. Overall, the results for glycerol were similar to the biomass results. T_4_ was the least (0.49 Pt; Fig. 6), while T_3_ was the most (2.34 Pt) impactful scenario. Although glycerol productivity was higher in T_3_ (7.39 mg L^−1^ d^−1^) than in T_1_ (4.28 mg L^−1^ d^−1^) and T_2_ (6.26 mg L^−1^ d^−1^; Table 3), it was not enough to compensate the impacts of MJM components. Glycerol productivity (*r* =  − 0.98, *p* = 0.003) and nitrogen concentration (*r* =  − 0.99, *p* < 0.001) were negatively correlated with damage results, while salt concentration was not correlated (*r* = 0.40, *p* = 0.51). As discussed above, NaCl is the main component of MJM contributing to impacts. This demonstrates that the linear correlation (*r*) is not enough to assess how the variables influence each other, and the LCIA is important to quantitatively identify a component’s contribution.

### Uncertainty analysis

Although LCA is a powerful tool for predicting the best scenarios and opportunities for improvement within a production chain, inaccuracies regarding the actual input values reflect on the output accuracy. If an LCA is expected to play a crucial role in decision-making, the quality and uncertainties of the results must be clear (Heijungs and Huijbregts 2004). These uncertainties propagate through the analysis and cannot be neglected, especially when many inputs are involved. The Monte Carlo simulation helps to assess the uncertainties of a given LCA scenario. It performs repeated interactions based on random input values within a specified probable range (Raynolds et al. 1999; Heijungs and Lenzen 2014).

S_3_, S_5_ (*D. salina* chain), and T_4_ (*D. tertiolecta* chain) were chosen for the uncertainty analysis using Monte Carlo simulation because they were the best scenarios predicted by the LCA. As shown in Table 5, uncertainties were similar between the three scenarios. The variation coefficient (VC), i.e., the uncertainty, was higher for “ionizing radiation” (82.3–91.7%), “non-carcinogens” (65.0–69.0%), and “aquatic eutrophication” (50.5–65.8%), demonstrating that imprecision was high for the inputs that contributed to these categories. “Respiratory inorganics,” “Non-renewable energy,” and “Global warming,” the three most impacted categories, presented the VC = 10.4–11.7%. The interpretation of a Monte Carlo simulation depends on the critical appreciation of the LCA practitioner; that is, conclusions are drawn based on the knowledge and judgment of those who analyze the results. However, the IPCC (2022) states that a VC of less than approximately 30% is considered reasonable. Thus, LCA predicted the three best scenarios with acceptable accuracy.

**Table 5:** Variation coefficient (%) resulting from the uncertainty analysis using Monte Carlo simulation (normal distribution, 1000 interactions, 95% confidence) for the proposed best scenarios

Impact categories	*D. salina*		*D. tertiolecta*
	S_3_	S_5_	T_4_
Carcinogens	43.2	40.3	42.0
Non-carcinogens	65.0	69.0	68.1
Respiratory inorganics	**11.4**	**11.2**	**11.7**
Respiratory organics	**10.8**	**10.6**	**10.8**
Ionizing radiation	91.7	82.6	82.3
Ozone layer depletion	**19.2**	**19.2**	**19.3**
Aquatic ecotoxicity	41.6	37.8	44.5
Terrestrial ecotoxicity	38.2	38.7	44.0
Terrestrial acidification/nutrification	**10.5**	**9.5**	**10.2**
Land occupation	**19.8**	**17.3**	**20.0**
Aquatic acidification	**13.6**	**11.6**	**12.9**
Aquatic eutrophication	50.5	65.8	52.4
Global warming	**10.4**	**10.7**	**10.9**
Non-renewable energy	**11.1**	**11.3**	**11.6**
Mineral extraction	**12.3**	**11.1**	**11.3**
Average (± standard deviation)	29.9 ± 23.8	29.8 ± 24.0	30.1 ± 22.9

Values in bold represent reasonable variation coefficient (< 30%) according to Pörtner et al. (2022)

**Fig. 7:**
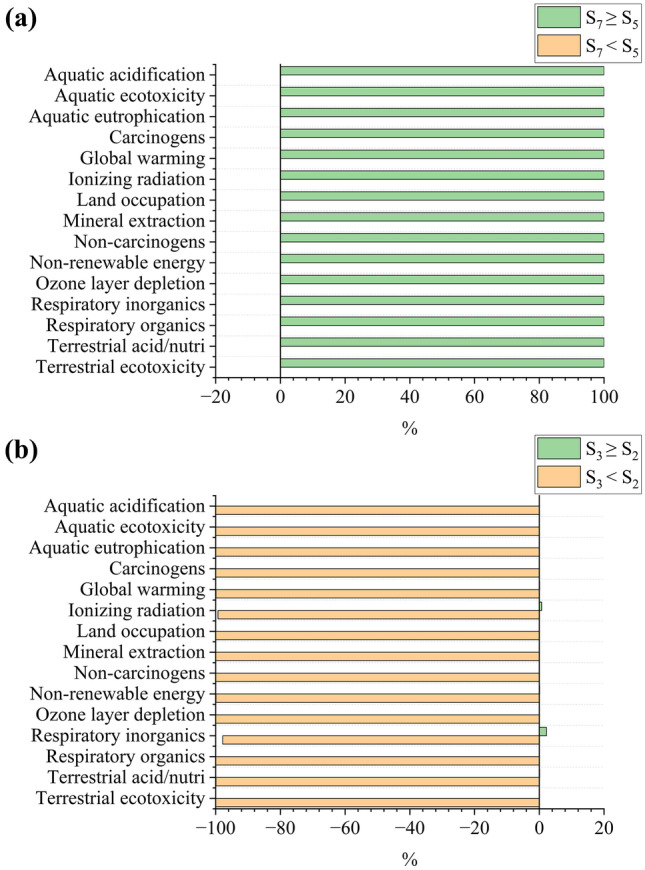
Uncertainty analysis comparing the best-predicted scenarios for biomass/glycerol (**a**) and beta-carotene (**b**) produced by *D. salina*. **a** represents the production of biomass and glycerol as they had the same results. Terrestrial acid/nutri = terrestrial acidification/nutrification. “A” < “B” = the cases in which scenario “A” was better than “B”; “A” ≥ “B” = the cases in which scenario “A” was not better than “B”

**Fig. 8:**
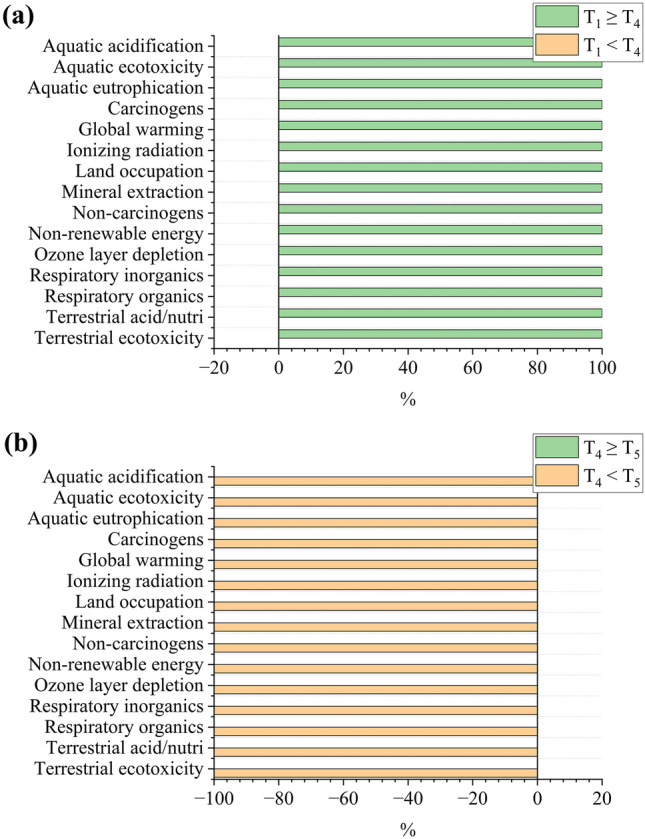
Uncertainty analysis comparing the best-predicted scenarios for biomass (**a**) and glycerol (**b**) produced by *D. tertiolecta*. **a** represents the production of biomass and glycerol as they had the same results. Terrestrial acid/nutri = terrestrial acidification/nutrification. “A” < “B” = the cases in which scenario “A” was better than “B”; “A” ≥ “B” = the cases in which scenario “A” was not better than “B”

To analyze the quality of the LCA in predicting the best scenarios considering the role played by uncertainties, a comparison was performed using Monte Carlo simulation to compare the two best-predicted scenarios for each bioproduct. Regarding biomass and glycerol production by *D. salina* (Fig. 7a), S_5_ had a minor impact for all categories in 100% of cases (i.e., interactions) simulated by the Monte Carlo. For beta-carotene (Fig. 7b), S_2_ had a lower impact in 2.2% of cases for “respiratory inorganics,” 0.7% for “ionizing radiation,” and 0.1% for “global warming,” “non-renewable energy,” and “terrestrial acidification/nutrification;” that is, S_3_ showed less uncertainty for beta-carotene production by *D. salina*. T_4_ was the best scenario in 100% of cases for all impact categories for biomass and glycerol production by *D. tertiolecta* (Fig. 8a, b). These results demonstrate that the uncertainties were not impactful enough to discredit the LCA predictions: S_5_ and S_3_ are the best scenarios for biomass/glycerol and beta-carotene production by *D. salina*, respectively, and T_4_ is the best scenario for biomass and glycerol production by *D. tertiolecta*.

### Perspectives and replicability of best scenarios

As demonstrated for the two analyzed chains (*D. tertiolecta* and *D. salina*), salt and deionized water had pronounced contributions to the LCA; however, environmental impacts can be significantly reduced by using brine from desalination plants and recycling the cultivation medium (Thomassen et al. 2018; Yildirim et al. 2022). Keller et al. (2017) calculated that the environmental impact contribution of brine to cultivate *D. salina* could be reduced by 99% by integrating *D. salina* cultivation with salt production or seawater desalination facilities. This is similar to our results: considering that brine from desalination plants could be used to supply salt for the cultivation of *D. salina* and *D. tertiolecta* (considering the productivity would be maintained), a reduction of over 69% in the damage categories could be obtained (Supplementary Material in the “*D. salina* – Impact” tab). Furthermore, combining species cultivation with flue gas mitigation can lessen the impacts associated with carbon supplementation, improve the MJM footprint, and reduce global warming (Collet et al. 2011). Wastewater can be used as an alternative culture medium to supply nutrients (de Souza Schneider et al. 2018), combining wastewater treatment and biomass yield (Celente et al. 2022); however, microalgae cultivation in wastewater limits biomass applications, especially in the food context.

Microalgae cultivation aiming at a single bioproduct yield has limited economic success (Merz et al. 2023). The co-production of multiple products could expand the applications of these species’ biomass (e.g., food, feed, and biofuel), leading to a system with lower environmental and economic impacts (Sui and Vlaeminck 2020). For example, Keller et al. (2017) reported that the co-production of zeaxanthin, all-trans beta-carotene, and lutein, among other bioproducts, reduced 0.5 t CO_2_ eq kg *9-*cis beta-carotene^−1^ (around 2% reduction) in a *Dunaliella*-based biorefinery. In our experiments, cultivating a higher nitrogen concentration resulted in higher biomass and glycerol productivity by *D. salina*, decreasing the overall impact. Nitrogen and protein accumulation are positively related (Uriarte et al. 1993; Sui et al. 2019), meaning the best scenario for biomass and glycerol production applies to protein yield. On the other hand, carbohydrate and lipid accumulation in *Dunaliella* spp. are increased under nitrogen-limited conditions (Uriarte et al. 1993; Yuan et al. 2019); thus, beta-carotene could be associated with the production of carbohydrates and lipids. While glycerol, protein, and beta-carotene can be used for food applications (Morrison 2000; Sui and Vlaeminck 2020), the remaining biomass can be used for biofuels (Karpagam et al. 2021): Mohamed et al. (2023) co-pyrolyzed sewage sludge with lignocellulosic and algal biomass to produce liquid and gaseous fuel, which significantly reduced global warming potential and achieved a high net positive energy balance.

Different parameters and cultivation protocols affect productivity, such as light intensity (Yuan et al. 2019), temperature (Mixson Byrd and Burkholder 2017), and growth phase (Sui et al. 2019), which were not in this scope. This shows that there is room for improvement in terms of optimizing the cultivation process. However, a new LCA would be required to predict the impacts and bottlenecks of the optimized system. In addition, energy must be considered when considering other variables unrelated to the medium composition, as exemplified by other authors (e.g., Hossain et al. (2019) and Porcelli et al. (2020)). For instance, Pérez-López et al. (2014) compared using natural sunlight and artificial lighting to cultivate *Haematococcus pluvialis* for astaxanthin production: sunlight greatly reduced the environmental impacts; however, lower productivity limited system improvement. Another crucial factor is the structure used to cultivate microalgae, such as raceways, open ponds, and photobioreactors, which not only affect productivity but also present different contributions to the environmental impact associated with materials. In this regard, novel and alternative materials are proposed to reduce costs and environmental impacts: for instance, Merz et al. (2023) repurposed and reused commercially available air-cushion packaging material as a low-cost, low-labor, and contamination-free photobioreactor (PBR) to cultivate *C. vulgaris*, *Nannochloropsis oculata*, and *Cyclotella cryptica* to produce biomass, lipid, and fucoxanthin with productivity compared favorably with traditional PBR. This not only offers an alternative to expensive conventional PBR but expends the lifetime of a material that otherwise would become waste (da Silva et al. 2021). The structure contribution to the LCA was not in the scope of our study.

Certainly, microalgal cultivation performance for bioproduct yield depends on complex biological, technological, physical, and geographic interactions (Jouannais et al. 2022), which does not always result in being environmentally friendly (Keller et al. 2017). Moreover, local legislation and the energy matrix also dictate microalgal bioproducts’ economic and environmental characteristics. Thus, the path from biomass quality identification to large-scale production is highly uncertain (Jouannais et al. 2022). Experimental data were obtained from the cultivation of the two species in Erlenmeyer flasks, which does not mimic real large-scale cultivation, for example, raceways PBRs. However, biomass productivity (58–133 mg L^−1^ d^−1^; Tables 2 and 3) used for this LCA was similar to previously reported for larger cultivation systems using artificial medium and could potentially provide comparable indications: García-González et al. (2005) produced 80 mg L^−1^ d^−1^ dry biomass of *D. salina* in f2 medium in an outdoor 55-L PBR; Kim et al. (2012) obtained 245 mg L^−1^ d^−1^ and 109 mg L^−1^ d^−1^ cultivating *D. salina* and *Dunaliella* sp. in D medium in a 12-L PBR; Zhu and Jiang (2008) produced 71 mg L^−1^ d^−1^ cultivating *D. salina* in an artificial medium in a PBR.

The data used for this LCA should not be used to calculate absolute impacts for large-scale cultivation of the two evaluated species; however, comparing these LCA results with large-scale production is still valid. The goal was to predict the best scenario regarding the composition of the MJM to produce biomass and bioproducts, which can be safely extrapolated to a large-scale cultivation system. Even if brine from a desalination plant were to be used, the contribution of each category to the total impact and damage score would be similar to the one identified in our proposed scenarios (Supplementary Material, “Damage and impact results” tab).

### Carbon footprint assessment

Global warming has been reported as one of humanity’s biggest problems (Acién Fernández et al. 2012): it has not only environmental impacts (Yoro and Daramola 2020) but also threats to human health and economic dynamics. As carbon dioxide is the main component of greenhouse gases (GHG) (Li et al. 2021), seeking more eco-friendly alternatives is a must to ensure the sustainability of our planet. Yadav et al. (2020) demonstrated through an LCA that the *Chlorella vulgaris* cultivation step (raceway open pond) was responsible for > 75% of the environmental impact related to GHG emissions. Although microalgae cannot be considered a tool to sequester CO_2_ as their biomass cannot store it for a long period, they can capture approximately 1.83 g of CO_2_ per g of biomass (based on a 40% carbon content in DW) (Acién Fernández et al. 2012), making them an important vector in the carbon flow.

**Fig. 9:**
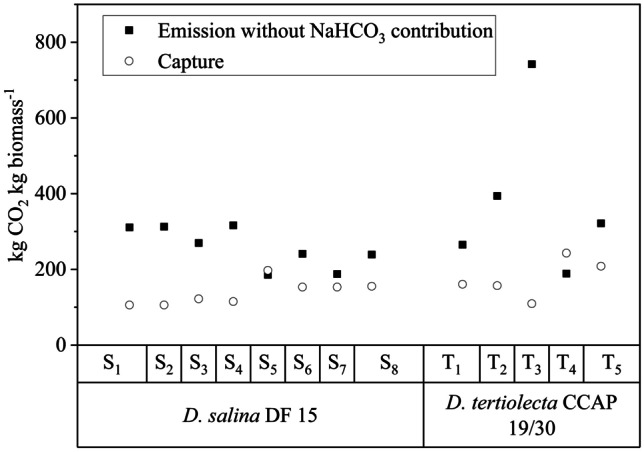
CO_2_-eq emission from the LCIA and the hypothetical CO_2_ captured by *D. salina* and *D. tertiolecta* for the proposed scenarios

Inorganic carbon as NaHCO_3_ was used to cultivate both species in our experiments. However, we have demonstrated that *D. tertiolecta* can grow on atmospheric carbon as efficiently as on NaHCO_3_ (Celente et al. 2022). Yadav et al. (2020) increased *C. vulgaris* biomass productivity by almost three times by supplying flue gas (CO_2_, 10% v/v). Thus, it is possible to assume that the productivity obtained in our experiments could be maintained if CO_2_ were to replace NaHCO_3_. Figure 9 shows the CO_2_-eq emissions concerning the MJM for the cultivation of the two species in different proposed scenarios and the hypothetical fixed CO_2_ (1.83 g CO_2_ g microalgal biomass^−1^ (Acién Fernández et al. 2012)) to obtain the same biomass productivity (Tables 2 and 3), considering that NaHCO_3_ was to be replaced by atmospheric CO_2_.

For *D. salina*, S_5_ presented negative carbon emissions (Δ =  − 12 kg CO_2_ kg biomass^−1^; Δ = kg CO_2_ emitted, kg CO_2_ captured), while S_2_ was the farthest (Δ = 207 kg CO_2_ kg biomass^−1^). In the case of *D. tertiolecta*, T_4_ showed negative CO_2_-eq emission (Δ =  − 54 kg CO_2_ kg biomass^−1^), demonstrating the potential of this species for a green economy. It is important to emphasize that our studies did not consider the impacts associated with energy consumption and infrastructure, which would contribute to more CO_2_ emissions and require further analysis. Pérez-López et al. (2014) demonstrated that electricity contributed to 61% of the GHG potential of *H. pluvialis* cultivation (considering the Belgium matrix, highly dependent on fossil fuel). Nevertheless, the energy impact directly depends on the supply matrix; thus, using energy from renewable sources can notably decrease the impact (Porcelli et al. 2020).

**Fig. 10:**
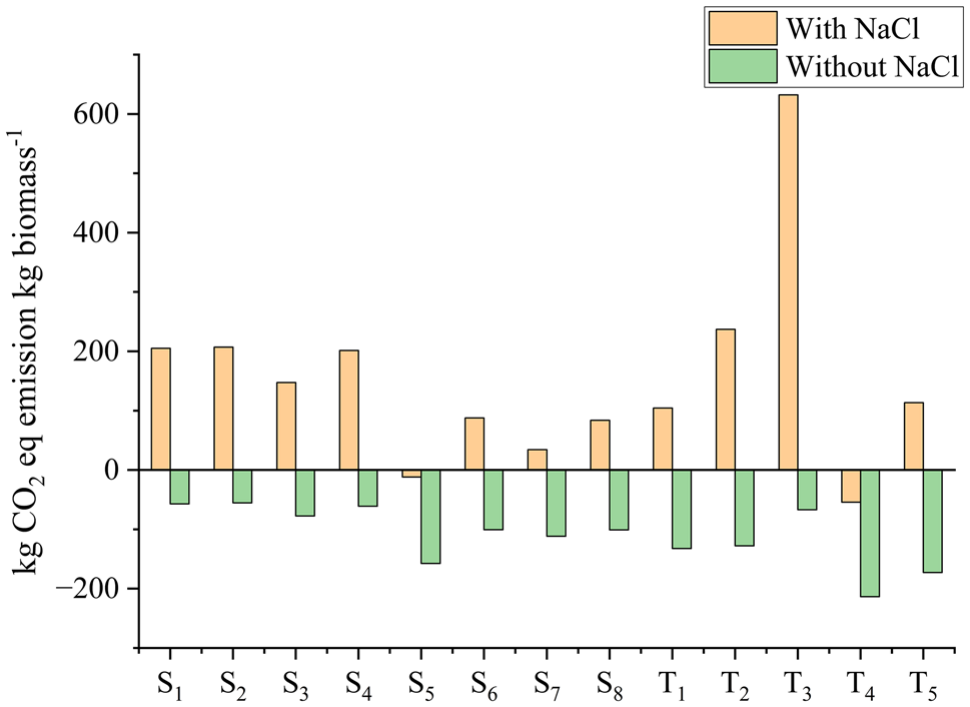
CO_2_-eq emissions by *D. salina* and *D. tertiolecta* for the proposed scenarios with and without NaCl contribution (assuming the use of brine from desalination plants). NaHCO_3_ contribution was neglected in both conditions (with and without NaCl contribution) as carbon would be supplied by CO_2_

However, if flue gas were to be used as an alternative CO_2_ source, it would further improve the carbon footprint. Yadav et al. (2020) reduced GHG emissions by approximately 45–50% GHG emissions when using flue gas from a thermal to cultivate *C. vulgaris* in open ponds compared to no carbon supply. Additionally, NaCl contributed to 70–90% of the CO_2_-eq emission (Supplementary Material, “*D. salina* – Impact” and “*D. tertiolecta* – Impact” tabs). Using NaCl as an input may have overestimated the environmental impacts for the proposed scenarios since obtaining sea salt (replacement in our experiments) does not require a purification step like NaCl. As demonstrated in the Sect. 3.4, NaCl significantly contributes to the LCA; if *D. salina* and *D. tertiolecta* were cultivated using brine from desalination plants, all the scenarios would present negative CO_2_ emissions (Fig. 10). CO_2_ emission would be reduced by 75%, 71%, and 78% for the best-proposed scenarios S_3_, S_5_ (*D. salina*), and T_4_ (*D. tertiolecta*), respectively, further improving carbon footprint (further information can be found in Supplementary Material, “CO2 – net” tab). However, it is important to highlight that the calculated emissions consider only the contribution of components in the artificial media and exclude other contributions, such as electricity, infrastructure, and other steps relevant to microalgae cultivation and bioproduct recuperation.

## Conclusions

The LCA was an important tool to accurately predict the best scenarios regarding the composition of the MJM for the analyzed bioproducts, proved by the Monte Carlo simulation. Although the data results from laboratory-scale experiments, the comparative LCA results still provide valuable indications for large-scale cultivation. Among the proposed scenarios for *D. salina*, S_5_ (1 g L^−1^ KNO_3_, 1.1 g L^−1^ MgCl_2_.6H_2_O) was the best in terms of environmental impacts for biomass and glycerol production, while S_3_ (0.1 g L^−1^ KNO_3_, 1.9 g L^−1^ MgCl_2_.6H_2_O) was the best for beta-carotene. T_4_ (1 g L^−1^ KNO_3_, 116.9 g L^−1^ sea salt) offered the best approach to cultivating *D. tertiolecta* for biomass and glycerol yield. When comparing both strains regarding biomass production, the best scenarios (S_5_ and T_4_) presented similar impact potential (approximately 74 mPt) as they presented similar productivities despite different medium compositions (refer to Tables 2 and 3). On the other hand, *D. tertiolecta* cleared performed better (damage score of 0.49 Pt for T_4_) than *D. salina* (0.95 Pt for S_5_) for glycerol production as the former produced almost 2.5 times more than the last (based on the best scenarios). Since *D. tertiolecta* does not produce significant amounts of beta-carotene, *D. salina* is the best candidate among the two species for this purpose. These results demonstrated that environmental impact assessment is not straightforward: increasing the nitrogen or magnesium concentrations does not necessarily result in more impact, as productivity can be significantly improved under these conditions and depends on the species. Overall, productivity could overcome the impacts of “surplus” artificial medium components. Our study also demonstrated the opportunity to combine *D. salina* and *D. tertiolecta* cultivation with CO_2_ capture and use brine from desalination plants, which helps to achieve an eco-friendlier cultivation system with a lower carbon footprint. Nevertheless, the LCA provides useful information for decision-making as the cultivation step is often the most impactful step for “Human health” and “Resources” due to its high contribution to “Respiratory inorganics” and “Global warming”.

## Supplementary Information

Below is the link to the electronic supplementary material.

Supplementary file1 (XLSX 115 KB)

## Data Availability

The datasets generated or analyzed during the current study are included in this published article and its Supplementary information file.
